# Digital light 3D printing of a polymer composite featuring robustness, self-healing, recyclability and tailorable mechanical properties

**DOI:** 10.1016/j.addma.2022.103343

**Published:** 2023-01-05

**Authors:** Wei Huang, Jianhui Zhang, Vikaramjeet Singh, Lulu Xu, Prasenjit Kabi, Eral Bele, Manish K. Tiwari

**Affiliations:** a Nanoengineered Systems Laboratory, UCL Mechanical Engineering, University College London, London WC1E 7JE, UK; b Wellcome/EPSRC Centre for Interventional and Surgical Sciences, University College London, London W1W 7TS, UK; c UCL Mechanical Engineering, University College London, London WC1E 7JE, UK

**Keywords:** DLP printing, Self-healing, Mechanically tailorable, Materials recycling, Lattice structures

## Abstract

Producing lightweight structures with high weight-specific strength and stiffness, self-healing abilities, and recyclability, is highly attractive for engineering applications such as aerospace, biomedical devices, and smart robots. Most self-healing polymer systems used to date for mechanical components lack 3D printability and satisfactory load-bearing capacity. Here, we report a new self-healable polymer composite for Digital Light Processing 3D Printing, by combining two monomers with distinct mechanical characteristics. It shows a desirable and superior combination of properties among 3D printable self-healing polymers, with tensile strength and elastic modulus up to 49 MPa and 810 MPa, respectively. Benefiting from dual dynamic bonds between the linear chains, a healing efficiency of above 80% is achieved after heating at a mild temperature of 60 °C without additional solvents. Printed objects are also endowed with multi-materials assembly and recycling capabilities, allowing robotic components to be easily reassembled or recycled after failure. Mechanical properties and deformation behaviour of printed composites and lattices can be tuned significantly to suit various practical applications by altering formulation. Lattice structures with three different architectures were printed and tested in compression: honeycomb, re-entrant, and chiral. They can regain their structural integrity and stiffness after damage, which is of great value for robotic applications. This study extends the performance space of composites, providing a pathway to design printable architected materials with simultaneous mechanical robustness/healability, efficient recoverability, and recyclability.

## 1 Introduction

Lattice architectures, ordered arrangement of struts or plates made of polymer composites that form the edges or faces of repeating unit cells, have found unprecedented prospects in aerospace structures, electronic devices, and biological tissues due to their multifunctionality and remarkable combination of mechanical properties and light weight [1], [2], [3], [4], [5], [6], [7], [8], [9]. Particularly, they are in great demand for load-bearing components (e.g., robotic arms) to avoid premature failure in severe conditions of loading [8], [10], [11]. In such components, the use of base materials with self-healing properties offers a more sustainable strategy to make things [12]. Self-healing polymers are able to repeatedly self-repair from physical damage by utilizing reversible interactions between the molecular components such as ionic bonding [13], [14], [15], [16], hydrogen bonding [12], [17], [18], [19], [20], metal-ligand coordination [19], [21], [22], [23], [24], [25], disulfide linkages [26], [27], [28], [29], and/or hydrophobic interactions [30]. This can extend their service life significantly; thereby, reducing overall maintenance expenses [12], [31]. However, fabricating healable polymers with high mechanical strength remains extremely challenging, because the diffusivity and exchangeability of dynamic polymer chains – the requirement for healing efficacy – come at the cost of mechanical strength where high chain rigidity and crystallinity are beneficial [17], [32], [33], [34], [35]. A vast majority of reported self-repairable polymers exhibit weak mechanical strength (less than 25 MPa) [17], [31], [36], [37], [38], [39], [40], making them unsuitable for applications such as aerospace industry[18], [37], [38]. Significant enhancement in the mechanical strength has been reported using hierarchical bonds [17], [36], [41], [42], [43], [44], multiphase designs [18], [45], and polymeric complexes [12], [16], [46], [47], [48]. However, the resulting composites require high temperatures (>90 °C), a long healing time of > 24 h [12], [31], and specific solvents [36] to activate/initiate the self-healing function [12], [17], [41], [42]. For example, An et al. combined polyacrylic acid (PAA) and polyvinylpyrrolidone (PVPON) to fabricate the water-driven self-healing polymeric complexes with tensile strength up to 81 MPa where the total healing time was over 29 h [12]. By exploiting a transition of the amorphous phase, Eom et al. imparted high tensile strength (43 MPa) and solvent-free self-healing properties at 35 °C to repairable carbonate-based thermoplastic polyurethane (TPU) films [18]. However, the high-strength self-healing polymers they reported were typically formed and tested as thin films. The limited fabrication methods (typically casting and moulding) have restricted the application of these polymer composites in useful 3D architectures.

More recently, digital light processing (DLP) printing has emerged as a powerful strategy to manufacture complex polymeric 3D structures with customizable functionalities and high printing efficiency [49], [50], [51], [52], [53], [54]. DLP employs patterned light to polymerize liquid resin into a solid layer in situ. Notably, recycling printed architectures could significantly reduce environmental pollution and waste of resources, thereby decreasing the overall cost of fabrication [55], [56]. Bifunctional and/or multifunctional light-curable thermosetting resins being heavily used by DLP to reduce the printing time and eliminate mechanical degradation are facing a huge challenge in recycling [49], [50], [55], [57], [58]. Therefore, the development of recyclable polymer composites without compromising other functionalities is of substantial importance for the synthesis of multifunctional composites.

In an attempt to address the above-mentioned problems in 3D printed composites, we introduce rational modifications leading to recyclability and superior mechanical strength, while maintaining self-healing capability within a reasonable time and temperature treatment. The methodology relies on a hard-soft molecular segment design where 4-acryloylmorpholine (ACMO) is the rigid segment and monofunctional urethane (MUA) is the soft portion used to form the composite ACMO-Zn-MUA cross-linked by hydrogen bonding and ionic bonding. Among the various light-curable monomers used in DLP printing, monofunctional 4-acryloylmorpholine (ACMO) has numerous advantages such as lack of odour, efficient printability, and recyclability [49], [50], [59]. Particularly, in addition to having relatively stiff molecular chains required for high mechanical properties, it also has a large number of carbonyl groups that can form hydrogen bonds with other polymers such as polyurethane, which not only maintains their high mechanical performance but also has the potential for self-healing properties [50], [60]. Starting with ACMO as the base resin, we chose thermoplastic monofunctional urethane (MUA) as the complementary monomer, which avoids forming non-recyclable thermosets cross-linked by reticulated covalent bonds, thereby facilitating the recycling of printed items [49]. This approach is beneficial for reducing global plastic waste accumulation and promoting sustainability. Also, the presence of soft MUA chains along with hydrogen and ionic bonding eliminates brittleness by acting as energy-absorbing “springs” between the stiff ACMO segments, allowing for a significant increase in mechanical strength over the individual components. Meanwhile, the introduction of zinc methacrylate (ZMA) could endow the composite with a second dynamic bonding (ionic bonding) [55], thus contributing to the multi-material assembly and further self-healing performance. Moreover, the self-healing properties of this polymer composite were illustrated in the mechanical response of three common lattice architectures: honeycomb, re-entrant, and chiral. The rugged lattices we printed are capable of recovering their structural integrity and stiffness after a fracture. Experimental results also demonstrate that lattice structures using healable composites exhibited more global and “sticky” deformation patterns compared to conventional lattice architectures. This study introduced self-healing and 3D printing capabilities without compromising recyclability and strength, which not only addresses the trade-off between self-healing and mechanical properties but also enables efficient manufacturing and recycling of robust polymeric structures.

## 2 Materials and methods

### 2.1 Materials

4-acryloylmorpholine (ACMO) and monofunctional urethane (MUA) were selected as the monomers. Zinc methacrylate (ZMA) was introduced as a cross-linker to further improve the self-healing properties of the composite. MUA was supplied by Xinfeng Bossin Polymer Materials Co., Ltd., China. 4-Acryloylmorpholine (ACMO), Phenylbis(2,4,6-trimethylbenzoyl) phosphine oxide (Irgacure 819), Sudan III, and zinc methacrylate (ZMA) were all purchased from Sigma-Aldrich. Phthalocyanine blue 15 was bought from J&K Scientific Ltd., China.

### 2.2 Printing resin preparation

The printing resins were prepared by adding 1 g Irgacure 819 (photoinitiator), 1 g ZMA (cross-linker), and 0.025 g Sudan III or phthalocyanine blue 15 (photoabsorber) in 100 g ACMO/MUA mixture (the weight ratios of ACMO/MUA = 10:0, 8:2, 6:4, and 4:6). The mixture resins were stirred for 40 min at 50 °C to form a homogenous solution before printing. The dye-free resins were prepared in the absence of photoabsorber. The mechanical performance, self-healing efficiency, and recyclability of the printed composites were not affected by the photoabsorbers.

### 2.3 DLP 3D printing

**Fig. 1:**
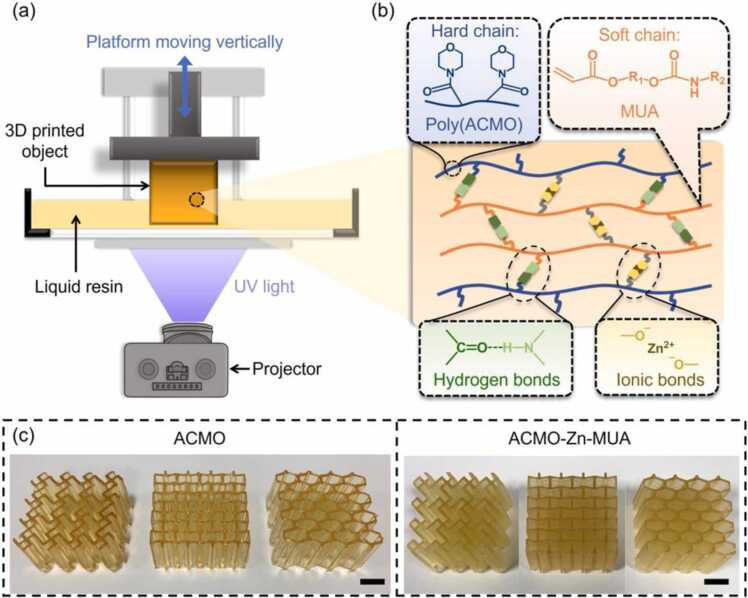
Design and rationale of recyclable photocurable resin with superior mechanical and self-healing properties. (a) Illustration of the DLP 3D printing setup. (b) Schematic depicting the dynamic interaction between the stiff (4-acryloylmorpholine (ACMO) in orange) and compliant (monofunctional urethane (MUA) in blue, R_1_ = (CH_2_)_*n*_, R_2_ = (CH_2_)_*n*_CH_3_, *n* = 2, 3, or 4) components via reversible hydrogen and ionic bonds resulting in both superior strength and self-healing within the printed composite. (c) (Left to right) Chiral, re-entrant, and honeycomb lattices printed using pure ACMO and ACMO-Zn-MUA resins. Scale bars are 1 cm.

Fig. 1(a) presents the DLP 3D printer used for printing the ACMO-Zn-MUA polymer composites consisting of ACMO and MUA as linear chains, zinc methacrylate (ZMA) as cross-linker, and Phenylbis(2,4,6-trimethylbenzoyl) phosphine oxide (Irgacure 819) as the photoinitiator. MUA monomer can only be used as an auxiliary resin for DLP printing. Our DLP printing set-up allows us to print liquid resins with MUA content up to 60 wt% due to its layer curing time limitation. Therefore, the composites with MUA contents from 0 to 60 wt% were printed and investigated in this work. The rheological properties of the mixed precursor resins play an important role in the quality and speed of DLP printing [61]. With each lift of the printing platform during the printing process, the liquid resins are required to be able to adequately fill the platform gap in preparation for the next layer of printing. Thus, the viscosity-shear rate curves were captured for characterizing the applicability of the liquid resins for printing. As shown in Fig. S1, the precursor resins with MUA concentrations ranging from 0 to 60 wt% exhibited viscosities of less than 60 mPa·s, making them ideal for commercial DLP printing [62]. It can also be seen that the viscosity of the liquid resins increased slightly with the increase of MUA content (Fig. S1). After photopolymerization, the liquid resin formed a mechanically strong ACMO-Zn-MUA polymer composite consisting of hard and soft linear chains linked by hydrogen bonds between C=O and N − H and ionic bonds between Zn^2+^ and −COO^−^ (Fig. 1(b)). Unlike the traditional thermosets printed with bifunctional or multifunctional monomers, the reversible bonds in thermoplastic polymers facilitate the repeated recyclability of the 3D printed objects. The glass transition temperature (*T*_g_) of the polymer composites with different MUA contents was obtained by differential scanning calorimetry (DSC) measurement as shown in Fig. S2. MUA monomer is soft with a *T*_g_ of only − 19 °C, while poly(ACMO) is a rigid polymer with a high glass transition temperature around 142 °C (see Fig. S2). Notably, two *T*_g_s can be identified in the DSC thermograms of ACMO/MUA blends, which can be ascribed to the two-component polymer system [63], [64]. Additionally, DSC results show that the *T*_g_s decreased with increasing MUA loading, suggesting the ACMO acts as an enhancer of the thermal properties of the ACMO/MUA composites [65].

Digital models were designed in the computer-aided design software SolidWorks. Each lattice has 4 × 4 unit cells with the same relative density (0.33) and overall size (40 mm × 40 mm × 20 mm). Their geometry and dimensions are shown schematically in Fig. S4. The commercial bottom-up DLP 3D printer with a 405 nm ultraviolet (UV) system was applied to produce the 3D printed objects. All printed models were generated with a 50 µm slice thickness. The UV exposure time for each layer was 8–12 s. After curing, the printed 3D samples were washed with ethanol and then post-cured in a UV chamber (405 nm) for 5 mins. Fig. 1(c) shows an array of printed ACMO-based lattices with diverse geometries (chiral, re-entrant, and honeycomb).

### 2.4 Recycling procedure

The printed objects were dissolved into an ACMO monomer at 140 °C. Then, Irgacure 819 (1 wt%) and ZMA (1 wt%) were added into the resulting liquid to produce the recycled printing resin. To obtain the recycling performance, printed dog-bone samples (ACMO/MUA = 8:2) were added into 80 g ACMO monomer under constant agitation at 140 °C until a homogeneous solution was made. Afterwards, 1 g Irgacure 819, 1 g ZMA, and 20 g MUA were added in the obtained solution under mixing at 50 °C for 40 min to form the recycled resin. Reprinted dog-bone samples were prepared from the recycled resin by the same DLP printer.

### 2.5 Mechanical test

Uniaxial tensile tests were conducted on an Instron machine (model 5969, equipped with 50 kN load cell) under a stretching speed of 1 mm/min at room temperature (23 °C), using DLP printed dog-bone specimen. The specimen geometry and dimensions are shown schematically in Fig. S5, which were in accordance with ASTM Type D 638 standards. Each test result was calculated by averaging data from three samples. The obtained forces were normalized by the original cross-sectional area to calculate the engineering stress, while the measured displacements were normalized by the original segment length to obtain the engineering strain. In-plane compression tests were performed on the DLP printed cellular composites (chiral, honeycomb, and re-entrant) at a loading rate of 5 mm/min. The toughness of the composites was calculated by integrating the area under the stress-strain curve.

Repeated Loading and Healing Test: The printed lattice was compressed to the first failure (loss of large load-bearing capacity for a short period of time) and then unloaded immediately. The sample with initial cracks was kept in the temperature chamber set at 60 °C for 6 h with the aim of healing. Before the next compression and healing cycle, each healed lattice was allowed to cool to the ambient temperature (23 °C). The loading and healing processes were repeated 4 times and 3 times, respectively (Fig. S6).

### 2.6 Characterization

The rheological properties of the liquid resins used for printing were measured using a Discovery Hybrid rotational rheometer (DHR3, TA instrument). The viscosities of the liquid resins before printing were measured at different shear rates (from 0.1 to 1000 s^−1^) under ambient conditions (23 °C). Differential scanning calorimetry (DSC) measurements were carried out on a Mettler Toledo DSC 3 STARe system instrument. The samples were heated from − 20–200 °C at a rate of 5 °C/min. Each measurement applied two heating-cooling cycles, and the data were acquired from the second heating process. Fourier transform infrared (FTIR) spectra of cured objects with various formulations were obtained using a spectrophotometer (Spectrum Two™, Perkin Elmer, ATR mode) in the region of 4000–600 cm^−1^, with a resolution of 4 cm^−1^. The fracture surface morphologies of dog-bone specimens were imaged using scanning electron microscopy (SEM) (EVO25, Carl Zeiss, Germany). For SEM observation, the specimens with suitable dimensions were fixed on the metal stake using double-sided adhesive carbon tapes and coated with a thin gold film, and then observed under an accelerating voltage of 5 kV. Thermal gravimetric analysis (TGA) was performed on a TA Instruments SDT 650 system from 40° to 500°C in a nitrogen environment at a heating rate of 10 °C/min.

## 3 Results and discussion

### 3.1 Mechanical response

**Fig. 2:**
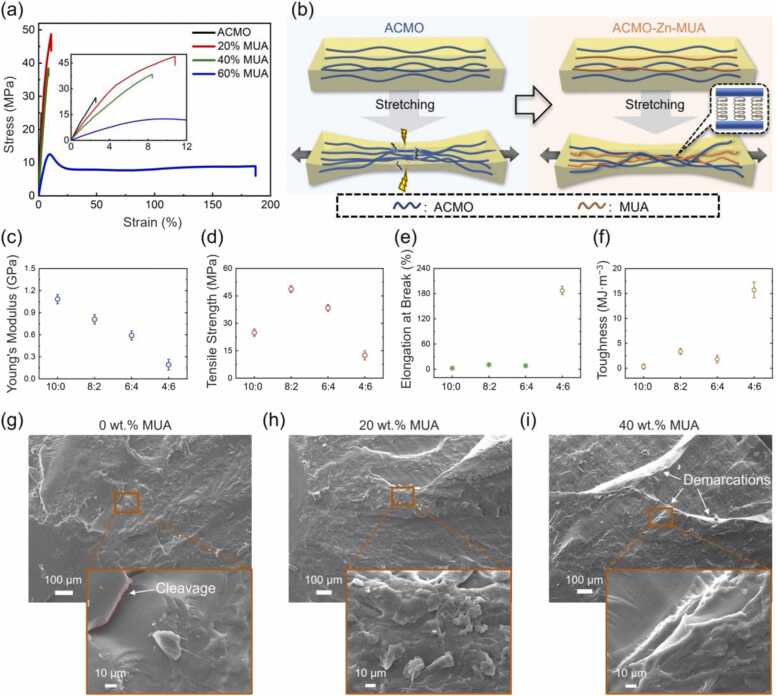
Mechanical characterization of the DLP printed ACMO-based composites. (a) Stress-strain curves of printed dog-bone samples with various MUA loadings (0, 20, 40, and 60 wt% of MUA) tested till failure. (b) Schematic illustration of the role of soft segments (MUA along with hydrogen and ionic bonding) in the sample under tension. (c) Elastic modulus, (d) ultimate tensile strength, (e) elongation at break, and (f) toughness of the dog-bone samples with different MUA loadings. Scanning electron microscopy (SEM) images of the fractured face of the (g) neat ACMO samples, (h) ACMO/MUA composites with 20 wt% MUA, and (i) ACMO/MUA composites with 40 wt% MUA.

Dog-bone shaped ACMO-Zn-MUA composites printed using DLP were tensile tested. The resulting stress-strain curves are presented in Fig. 2(a). Compared to neat poly(ACMO), Fig. 2(a) shows a significant increase in both tensile strength and elongation at break for the specimens containing 20–40 wt% of MUA. It is expected that the presence of soft MUA chains, hydrogen bonding, and ionic bonding eliminate brittleness by acting as energy-absorbing “springs” between hard ACMO segments (Fig. 2(b)). Thus, the combination of hard and soft segments in optimal proportions enables the ACMO-Zn-MUA composite to achieve an ultimate tensile strength of 48.7 MPa, which is higher than that of many commercial polymeric materials such as the soles used in footwear (43 MPa) [18]. The Young’s modulus for these samples is calculated from the initial slope of the stress-strain curve and shown in Fig. 2(c). The modulus clearly decreases with increasing MUA concentration. However, both the tensile strength and the elongation at break (Fig. 2(d, e)) decrease for 40 wt% MUA, which is similar to the trend for bicomponent polymers [66], [67], [68]. The decrease in strength could be due to excessive soft segments, making the composite compliant and weaker [66]. The reduction in elongation at break for higher concentrations of MUA is due to the destruction of the hard phase continuum [67]. At the highest MUA concentration of 60 wt%, the soft phase dominates, resulting in the lowest modulus but the highest toughness (Fig. 2(c, f)). The mechanical properties of the printed objects post-cured for different duration were compared to investigate the effect of post-curing time. No significant difference was recorded in their stress-strain curves as shown in Fig. S15. This suggests that the reduction in tensile strength and elastic modulus of the composite containing 60 wt% MUA is not due to incomplete curing but to the large volume of soft segment MUA.

The fracture surface morphology of samples was analysed using scanning electron microscopy (SEM) (Fig. 2(g-i)). The cleavage fracture surfaces confirm the intrinsic brittleness of neat poly(ACMO) (Fig. 2(g)) [60], while the grain morphology seen in Fig. 2(h) indicates that ACMO_0.8_-MUA_0.2_ developed a rough surface during stretching loading, dissipating a considerable amount of energy, which contributed to its superior mechanical properties (red curve in Fig. 2(a)) [69], [70]; For the ACMO_0.6_-MUA_0.4_, due to the presence of hard and soft segments with similar contents, a decrease in both tensile strength and elongation at break occurred compared to the ACMO_0.8_-MUA_0.2_ counterpart (Fig. 2(a)), which is similar to the results obtained with the combination of brittle poly(acrylamide) (PAAm) and elastic poly(acrylic acid) (PAA) in [67]. This can also be explained by the demarcations evident on their fracture surfaces, as shown in Fig. 2(i).

### 3.2 Self-healing performance and recyclability

**Fig. 3:**
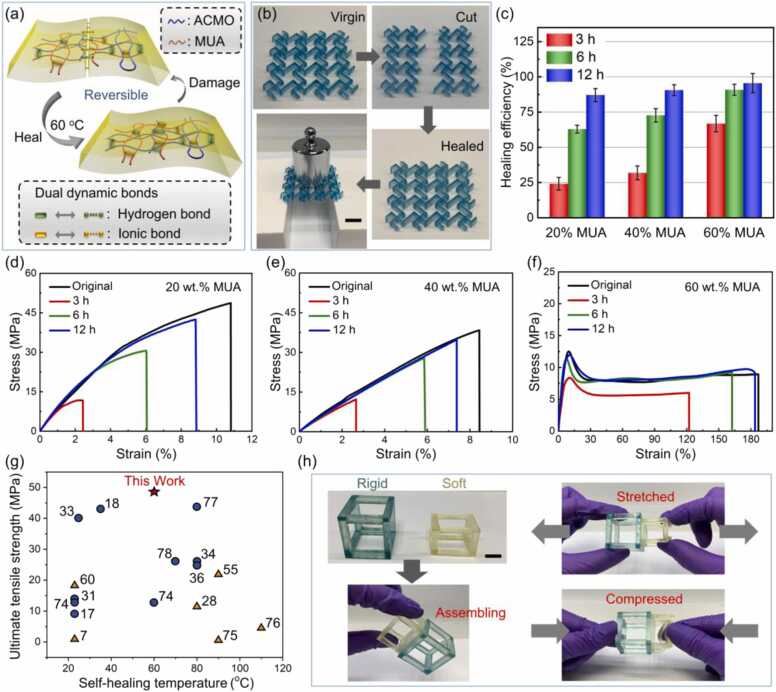
Self-healing and assembling performances of DLP printed ACMO-Zn-MUA composites. (a) Schematic illustration of self-healing properties functionalized by reversible hydrogen bonds (green cylinders) and ionic bonds (yellow cylinders). (b) Photographs of cut and healed lattice sheet with ACMO/MUA = 8:2, followed by a 100-g weight-support demonstration. (c) Self-healing efficiencies of the specimens with different formulations after healing at 60 °C for different time. The stress-strain curves of printed composites with (d) 20 wt%, (e) 40 wt%, and (f) 60 wt% MUA after healing at 60 °C for various time. (g) Ashby chart of tensile strength versus self-healing temperature of ACMO-Zn-MUA and various self-healing polymers reported in the literature. The red star denotes the properties of ACMO-Zn-MUA, the yellow triangles show the performance of the self-healable polymers with 3D printability, and the blue circles denote the performance of the healable films. (h) The assembling of a multi-material structure with rigid (ACMO/MUA = 8:2, blue hollow cube) and soft composites (ACMO/MUA = 4:6, yellow hollow cube). Scale bar equals 1 cm.

The self-healing property of ACMO-Zn-MUA can be attributed to the dual dynamic bonds within resulting composites (Fig. 1(b) and Fig. 3(a)). In addition to the hydrogen bonding between the urethane chains, there are reversible linkages between poly(ACMO) and MUA chains. Meanwhile, due to the introduction of zinc ions, the hard-soft chains were also cross-linked by ionic bonds, thus ensuring excellent self-healing efficiency. As shown in Fig. 3(b), a rigid lattice sheet (ACMO/MUA ratio at 8:2) was completely cut into two parts, then joined together and healed at 60 °C for 3 h. The healed sheet can support a 100 g weight without any damage or buckling. In order to quantitatively measure the self-healing efficiency of ACMO-Zn-MUA, defined as the ratio of the tensile strength of healed and virgin specimens, tensile tests were carried out on healed dog-bone samples with various compositions. First, the printed dog-bone samples were cut in half down the middle, then they were brought together and finally allow to heal by mild condition heating at 60 °C for varying amounts of time. Representative stress-strain curves of original and healed ACMO-Zn-MUA composites are shown in Fig. 3(d, e, f). The mechanical properties including tensile strength and toughness gradually recovered with the increase in healing time (Table S1). Fig. 3(c) also indicates that the healing efficiencies increased with healing time. For example, the tensile strength recoveries for the sample with the ACMO/MUA ratio at 4:6 are 66%, 91%, and 95% after 3, 6, and 12 h, respectively. As shown in Fig. S7, the mechanical properties of the samples healed at 30 °C for 12 h were inferior when compared to those healed at higher temperatures. Healing at 60 °C could largely restore their original mechanical strength, although their failure strain is smaller compared to the samples healed at 90 °C (Fig. S7, Table S2). Additionally, their healing efficiencies are strongly correlated with the concentration of MUA (Fig. 3(c)). This can be validated by the Fourier transform infrared (FTIR) spectroscopy tests (see Fig. S8) since FTIR spectroscopy is sensitive to hydrogen bonds [68], [71]. Fig. S8(a) shows a corresponding increase in the peak intensity of N − H urethane stretching with the increase of MUA content, indicating the improved self-healing efficiency due to the presence of hydrogen bonds [72]. Moreover, from the wavenumber range from 1780 to 1670 cm^−1^ shown in Fig. S8(b), the increase in the MUA loading transfers the stretching of the C=O group to a lower wavenumber due to the existence of hydrogen bonds between the C=O and N − H groups [71], [72]. The self-healing behaviour of the polymer composites with ZMA and without ZMA was compared. As shown in Fig. S9, the healed composites containing ZMA outperformed the healed samples without ZMA in terms of tensile strength and failure strain, indicating the role of dynamic ionic bonding in enhancing the self-healing properties of the composites. The ACMO-Zn-MUA composites manifested such efficient healing in the absence of external force, light, and solvents. In effect, the healing efficiency of above 80% was reached after healing at 60 °C for 12 h. For comparative assessment of the polymer composite presented here against those in literature, we created an Ashby diagram of tensile strength versus healing temperature as shown in Fig. 3(g). The performance of ACMO-Zn-MUA is superior when compared to contemporary self-healing polymers [7], [17], [18], [28], [31], [33], [34], [36], [55], [60], [73], [74], [75], [76], [77], since it achieves a high tensile strength and healing efficiency at a mild healing temperature of 60 °C (Fig. 3(g)). The thermal stability of the composite materials was obtained using a thermogravimetric analysis (TGA) system. The developed composite materials possess excellent thermal stability and the obtained data (Fig. S3) suggests that the composites are suitable for a wide range of engineering applications such as biomedical devices, intelligent robots, and certain aerospace [78].

Printing large-sized 3D objects and multi-materials has always been a stiff challenge in the field of DLP printing. Although a strategy of replacing resins can be performed to achieve multi-materials printing [79], tedious steps such as removing and washing the residual resin were required during printing. Given the mild healing temperature and high healing efficiency, multiple parts with distinct stiffness can be assembled, thus circumventing the size constraint of 3D printing. Fig. 3(h) shows a complex 3D structure assembled with rigid (ACMO/MUA = 8:2) and soft composites (ACMO/MUA = 4:6). They can be stretched and compressed and exhibit distinct deformation states as will be discussed in detail later.

**Fig. 4:**
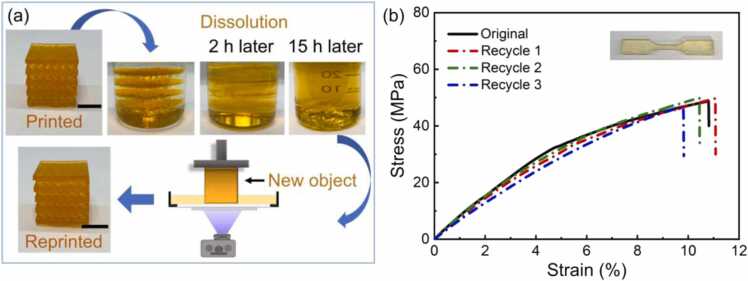
Recycling DLP printed ACMO-Zn-MUA. (a) The dissolution and reprinting process of printed 3D objects. (b) Stress-strain curves of initial and reprinted ACMO-Zn-MUA dog-bone samples with ACMO/MUA = 8:2. Scale bars are 1 cm.

For the recyclability of DLP printed parts, traditional multifunctional resins, even some monofunctional monomers such as acrylic acid (AA) and methyl methacrylate (MMA), cannot be employed due to challenges of solubility, suitable viscosity, and rapid polymerization rate [50]. Table S4 also lists and compares the maximum tensile strength, self-healing performance, and recyclability of the photocurable polymers reported in the literature. It is acknowledged that there are significant difficulties in developing printable polymers with simultaneous mechanical robustness, efficient self-healing ability, and recyclability. Herein, we exploited the solubility of the printed objects in a liquid monomer and the presence of reversible cross-linking, to demonstrate recyclability. The homogeneous solution obtained by dissolution in monomer was printed to demonstrate recyclability without degrading mechanical performance. Fig. 4(a) shows the printed objects can be dissolved in liquid ACMO liquid at 140 °C after 15 h and the resulting recycled resin can be used as resin ink for DLP. To quantitatively verify recycling performance, dog-bone samples were reprinted and tested up to three times. The stress-strain curves of ACMO-Zn-MUA dog-bone samples before and after recycling were compared and displayed in Fig. 4(b). The comparison is important as structural alternation is true for any recyclable polymer composition and it is critical to assess the change in the mechanical properties after the recycling process [80]. All samples showed comparable mechanical properties, with a tensile strength of around 48.8 MPa and a fracture strain of ∼10.6% (Table S3). Both the tensile strength and failure strain degraded by less than 10% after 3 cycles, proving the recycling effectiveness of the proposed polymer composite. Additionally, the recyclability of ZMA-free composites was evaluated and compared with the ACMO-Zn-MUA composite. It is important to note that although ZMA-free composite can be recycled and reprinted, the mechanical properties of the recycled samples degraded significantly when compared to those from original ZMA-free specimens (Fig. S10).

### 3.3 Structural response of lattice architectures

**Fig. 5:**
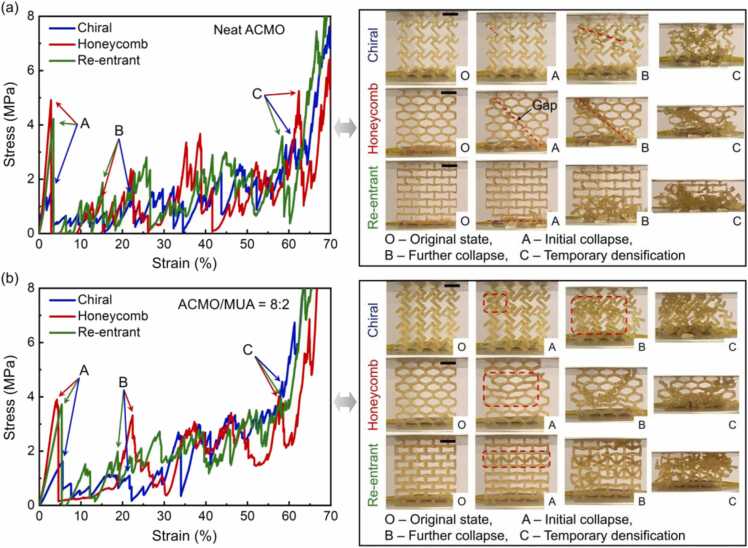
Continuous compressive stress-strain curves and corresponding deformation behaviours for printed (a) neat ACMO and (b) ACMO-Zn-MUA lattices with chiral, honeycomb, and re-entrant cell topology. Zoomed deformation images and videos can be found in the Supplementary Material (Fig. S11 and Video S1-S6). Scale bars are 1 cm.

Lightweight cellular composites with functionality have been popular for decades, yet efforts on the lattices made of self-healing composites are rare [7], [59]; reports to date have been limited to the demonstration of self-healing sensing behaviour and reversible deformation of compliant structures. There is still a lack of reports on the healing effectiveness of cellular composites for structural capacity, and the influence of the healing constituents (concentration) on the collapse response of stiff structures has also received limited attention. In this section, the failure response of ACMO-based composites shaped into three different cellular topologies was investigated. In-plane compression tests were performed on DLP printed ACMO and ACMO-Zn-MUA lattice structures with chiral, honeycomb, and re-entrant cell topology. Fig. 5(a) and (b) show the compressive stress-strain curves of neat ACMO lattices and ACMO-Zn-MUA lattices, respectively. Additionally, the compression process is recorded in the video, and the key events are presented as snapshots in Fig. 5(a) and (b). Their deformation responses display three sequential stages of initial collapse, further collapse, and temporary densification, which corresponds to the points labelled A, B, and C on the stress-strain plot, respectively. For point A in Fig. 5(a), all ACMO samples underwent an initial fracture involving a localized region (marked with red dotted lines in Fig. 5(a) and Video S1–3). Compared to chiral structure, honeycomb and re-entrant lattices exhibited larger stress peaks due to a higher number of collapsed struts. Notably, the fracture pattern observed in the honeycomb structure is the diagonal shear fracture (Fig. 5(a) and Video S2) associated with the lower ductility of the cellular composites [81], [82]. This resulted in the sharpest stress fluctuation among all lattices (see point A on the red line in Fig. 5(a)), suggesting that different cell topologies can trigger distinct collapse responses. Notably, the honeycomb lattice has no load-bearing capacity (between points A and B on the red line in Fig. 5(a)) due to the gap between the 45-degree fracture surfaces of the honeycomb under compression (the red dotted line on the honeycomb in Fig. 5(a) and Video S2). Thus, the toughness of the neat ACMO honeycomb lattice is lower than that of all other lattices (Table S6). When the strain was further increased, concentrated damage occurred in all cellular structures, followed by strain hardening caused by temporary densification of the lattices (see point C in Fig. 5(a)). In general, neat ACMO lattices have lower compressive strength and toughness compared to the ACMO-Zn-MUA lattices (Fig. 5 and Table S6), which could be attributed to the localized fracture (red dashed lines in Fig. 5(a) and Video S1–3) and the brittleness of the raw materials [81].

Conversely, the localized fracture phenomenon of neat ACMO lattices (marked with red dotted lines in Fig. 5(a)) is not observed for the ACMO-Zn-MUA cellular composites that deform more uniformly (marked with red dashed rectangles in Fig. 5(b) and Video S4–6) and exhibit improved toughness (Table S6), resulting in higher capacity to absorb energy. As shown in Fig. 5(b), the overall stress level in the stress-strain curves, especially in their later phase (after point B), is improved as compared to the lattices fabricated with pure ACMO, we can see that there is an average increase of 0.5 MPa. In addition, the toughness of the ACMO-Zn-MUA lattices increases by an average of 0.24 MJ·m^−3^ (∼22%) compared to the pure ACMO lattices (Table S6). The novel ACMO-Zn-MUA introduced here alleviates the brittleness in the bulk materials of lattices, as evidenced by a gentle increase in the modulus of elasticity at the outset of the stress-strain curves (before point A). It can also be seen from the deformation images that a more global and regular deformation pattern emerged upon compression. For the re-entrant lattice, the initial stress peak (point A) observed in the stress-strain curve is related to the rupture of the inclined ligaments within a horizontal row of cells (see image A of re-entrant lattice in Fig. 5(b) and Video S6). Upon further compression, more cell walls begin to contact with the adjacent struts, thus the more re-entrant cells stacked into the stretch-dominated triangular structures (Video S6), promoting an improved and stable mechanical response (between points B and C on the green line in Fig. 5(b)) compared to its ACMO counterpart (between points B and C on the green line in Fig. 5(a)). The re-entrant lattice outperformed all other lattices in terms of toughness due to its unique collapse response (Table S6). The chiral structure exhibited regular cell wall clustering upon compression (Video S4), accompanied by a significant improvement in mechanical properties, as opposed to pure ACMO lattices in which specific cells rotated clockwise (Video S1). This can be explained by the fact that the more ductile properties of the constituent materials (ACMO-Zn-MUA) allow the lattice structures to tolerate greater deformation without localized fracture, thus changing the deformation pattern of the lattices fabricated with ACMO-Zn-MUA. The honeycomb structure with ACMO-Zn-MUA also exhibited a relatively monolithic deformation pattern (Video S5) compared to the pure ACMO counterpart. Meanwhile, the mechanical properties and deformation behaviour of the lattice structures, honeycomb, made of the recycled composite material were recorded to be comparable to those produced from the original material (Fig. S12). Therefore, our approach of combining hard and soft segments eliminates the stress concentration occurring in neat ACMO lattices, which not only changes their collapse mode but also gives the lattice structures much improved mechanical properties compared to the conventional lattices.

Supplementary material related to this article can be found online at doi:10.1016/j.addma.2022.103343.

The following is the Supplementary material related to this article Video S1

Video S1.

Supplementary material related to this article can be found online at doi:10.1016/j.addma.2022.103343.

The following is the Supplementary material related to this article Video S2

Video S2.

Supplementary material related to this article can be found online at doi:10.1016/j.addma.2022.103343.

The following is the Supplementary material related to this article Video S3

Video S3.

Supplementary material related to this article can be found online at doi:10.1016/j.addma.2022.103343.

The following is the Supplementary material related to this article Video S4

Video S4.

Supplementary material related to this article can be found online at doi:10.1016/j.addma.2022.103343.

The following is the Supplementary material related to this article Video S5

Video S5.

Supplementary material related to this article can be found online at doi:10.1016/j.addma.2022.103343.

The following is the Supplementary material related to this article Video S6

Video S6.

**Fig. 6:**
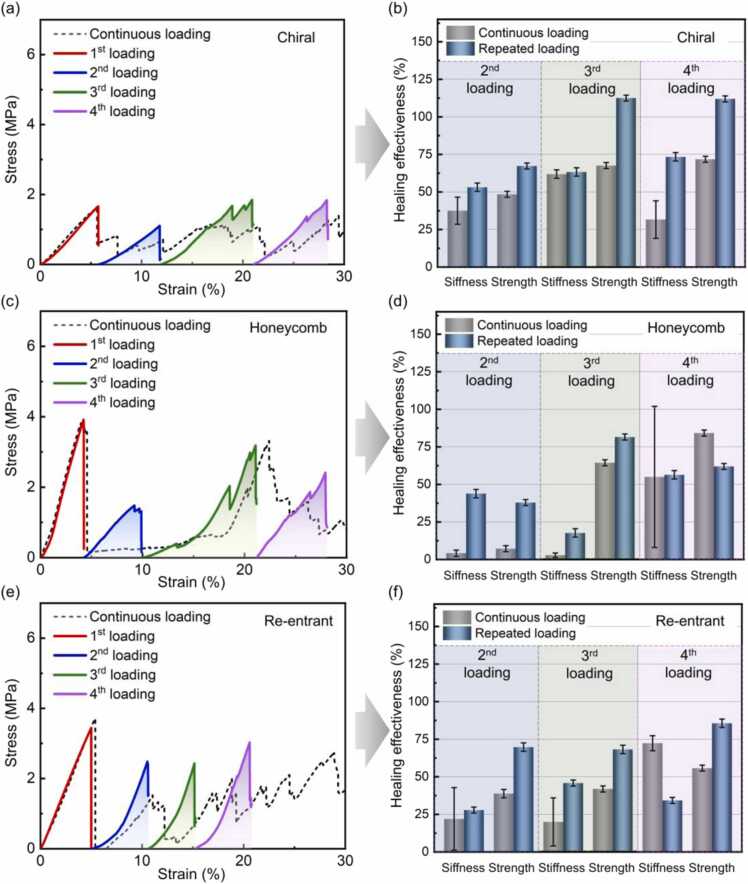
Comparison of continuous and repeated loading stress-strain curves for single (a) chiral, (c) honeycomb, and (e) re-entrant lattices, where the samples were allowed to heal at 60 °C and then cooled to 23 °C before the next test. Healing effectiveness of (b) chiral, (d) honeycomb, and (f) re-entrant lattice during different healing/loading cycles.

Besides the distinct characteristics under monotonic compression, the lattices fabricated with self-healing ACMO-Zn-MUA were also able to restore their structural integrity and stiffness after the initial fracture, which is of great importance for robotics [83]. To evaluate the healing effectiveness on structural capacity, repeated compression tests producing preliminary damage were performed on the three kinds of lattices that were healed between tests, as depicted in Fig. S6. Note that the cumulative compressive strain of repeated tests was controlled within 30% (see Fig. S13 and Fig. S14) that is meaningful for practical engineering applications because the cellular composites may lose their original architecture and dimensional accuracy under large deformations. The stress-strain curves of chiral, honeycomb, and re-entrant lattices under two modes of loading are compared in Fig. 6(a-f). And their toughness values calculated from each stress-strain curve are presented in Table S7. The structural healing effectiveness was calculated by normalizing the stiffness and peak stress of repeated loading by the initial stiffness and strength of the intact lattice, respectively. Different from the continuously loaded tests, repeated loading and healing operations result in superior mechanical properties including stiffness and strength over the same strain range (Fig. 6(a-f)), suggesting that the self-healing capability of the constituent materials can be exploited to maintain and recover the structural integrity (see Fig. S14), stiffness, and strength for repeated usage. In terms of the toughness of the lattices under compression, repeated loading and healing operations generally contribute to higher toughness values as compared to the continuous loading counterparts (see Table S7). Although a few of the repeated loading curves possess lower toughness values compared to the continuous loading (Table S7), the stress-strain curves for the repeated loading are steeper and more stable than those for continuous loading (Fig. 6(a, c, and e)). Additionally, the healing effectiveness is strongly correlated with the architecture of cellular composites. The healed chiral and honeycomb lattices show oscillations in the stress-strain curves near the stress peak during multiple loadings (see Fig. 6(a, c)), which is due to the fact that they are global bending dominated and repeated loading is more likely to break the microcracks generated by the previous loading, which in turn lead to multiple fractures and stress fluctuations. For the re-entrant lattices, the cell walls tend to deform into stable triangular structures upon compression, which results in a sharp increase in mechanical strength (Fig. 6(e, f)) and a slight decrease in cumulative compressive deformation (Fig. S14) due to the operation of self-healing between tests.

## 4 Conclusion

We have developed a rugged healable thermoplastic polymer composite for DLP printing. The hard-soft segment designed ACMO-Zn-MUA exhibited a tensile strength of 49 MPa, which is stronger than many commercial polymers. By changing the MUA loading, the elastic modulus, ultimate tensile strength, and failure strain of printed objects are highly tailorable, which may suit a variety of industrial requirements. Furthermore, by taking advantage of the dual dynamic bonds, efficient healing can function at 60 °C without the aid of light or additional solvents. The multi-materials assembly and printing were realized by utilizing their efficient healing ability. Moreover, the printed 3D objects could be recycled using the same DLP method to avoid resource wastage. Finally, the material-structure relationship of DLP printed cellular structures was systematically investigated and compared, and the results showed that our method of combining hard and soft phases significantly improves the collapse characteristics of the lattices, hence enhancing their mechanical properties. Meanwhile, the robust lattices made of self-healing composites could effectively recover their structural integrity and stiffness after damage. This study not only alleviates the conflict between strength and self-healing but also realizes efficient recycling and healing of rugged structures.

## CRediT authorship contribution statement

**Huang Wei:** Writing – original draft, Validation, Methodology, Investigation, Formal analysis. **Bele Eral:** Writing – review & editing, Supervision, Methodology. **Tiwari Manish K.:** Writing – review & editing, Visualization, Supervision, Resources, Project administration, Funding acquisition. **Zhang Jianhui:** Visualization, Investigation. **Singh Vikramjeet:** Writing – review & editing, Supervision, Methodology. **Xu Lulu:** Writing – review & editing, Investigation. **Kabi Prasenjit:** Writing – review & editing, Methodology.

## Declaration of Competing Interest

The authors declare that they have no known competing financial interests or personal relationships that could have appeared to influence the work reported in this paper.

## Data Availability

Data will be made available on request.
