# Supplementary material for: Digital light 3D printing of a polymer composite featuring robustness, self-healing, recyclability and tailorable mechanical properties
Source: Addit Manuf. 2023 Jan 5;61:None. doi: 10.1016/j.addma.2022.103343 (PMC10567580; doi:10.1016/j.addma.2022.103343)
Supplement: Supplementary file 7 — Supplementary material [file mmc1.docx]

Supplementary Material

Digital light 3D printing of a polymer composite featuring robustness, self-healing, recyclability and tailorable mechanical properties

Wei Huang^a^, Jianhui Zhang^a^, Vikramjeet Singh^a^, Lulu Xu^a,b^, Prasenjit Kabi^a^, Eral Bele^c^, Manish K. Tiwari^a, b,^ *

^a^Nanoengineered Systems Laboratory, UCL Mechanical Engineering, University College London, London, WC1E 7JE, UK.

^b^Wellcome/EPSRC Centre for Interventional and Surgical Sciences, University College London, London, W1W 7TS, UK

^c^UCL Mechanical Engineering, University College London, London, WC1E 7JE, UK.

***Corresponding author. E-mail:** [**m.tiwari@ucl.ac.uk**](mailto:m.tiwari@ucl.ac.uk)

**
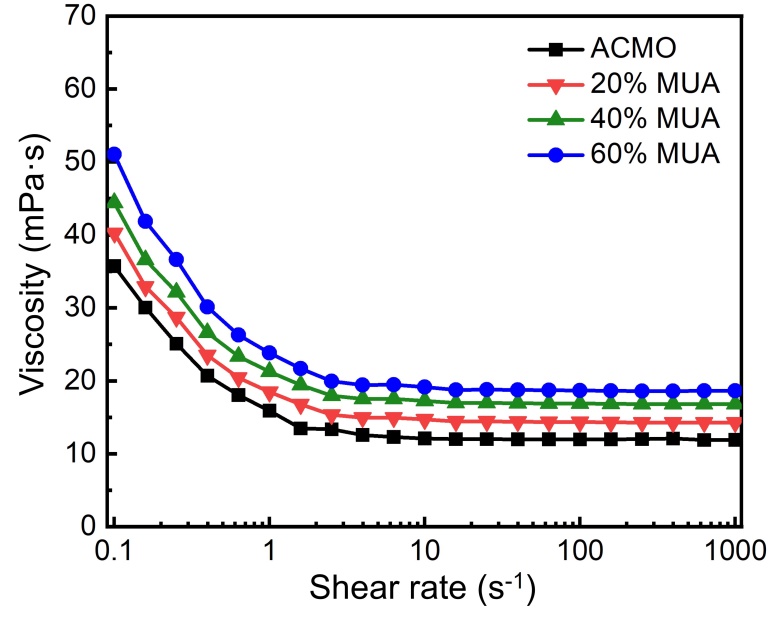
**

**Fig. S1.** Viscosity-shear rate curves of the liquid resins with different MUA loadings (0 wt.%, 20 wt.%, 40 wt.%, and 60 wt.%).


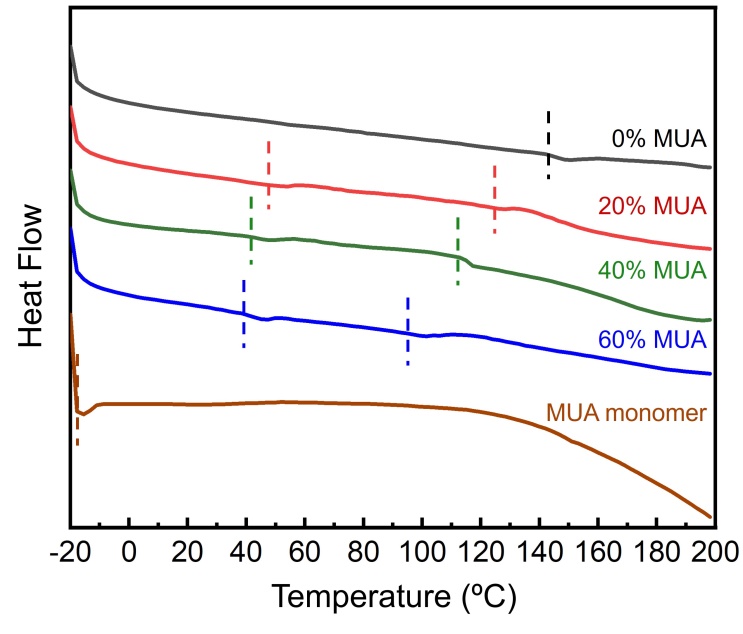


**Fig. S2.** DSC curves of the composites with different MUA loadings.


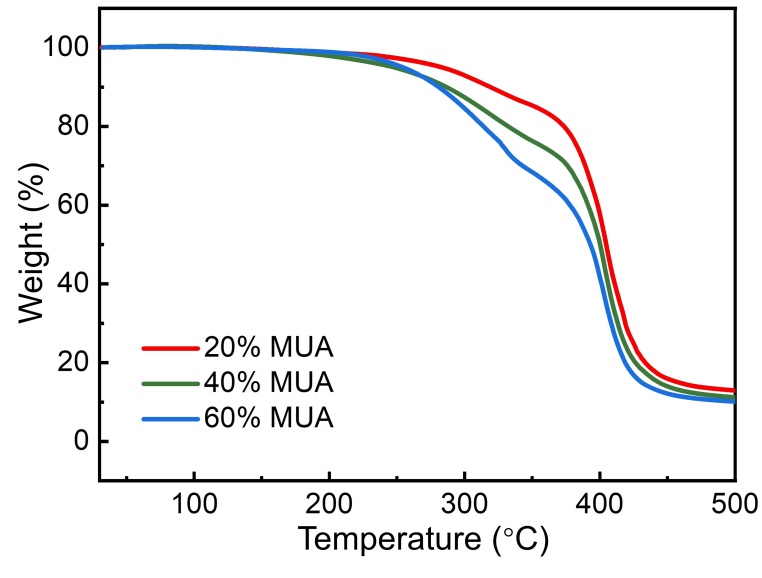


**Fig. S3.** TGA of the composite materials with different MUA loadings.


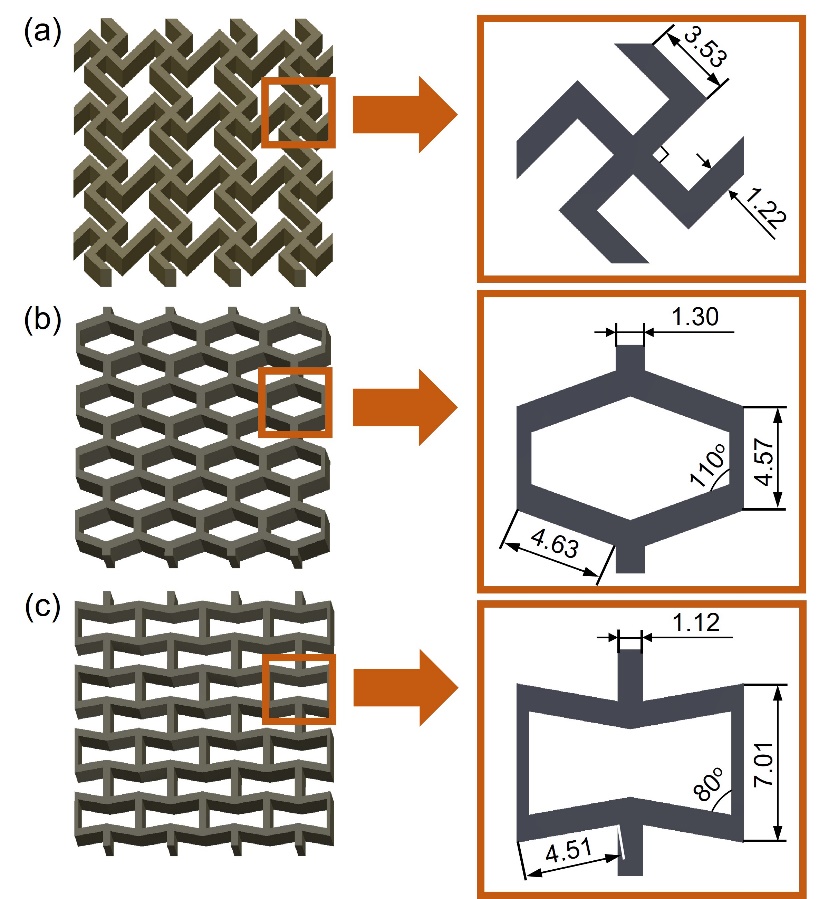


**Fig. S4.** Geometric parameters of (a) chiral (top row), (b) honeycomb (middle row), and (c) re-entrant (bottom row) lattices, all of them have an equal relative density of 0.33. Dimensions are in mm and the specimen thickness is 20 mm.


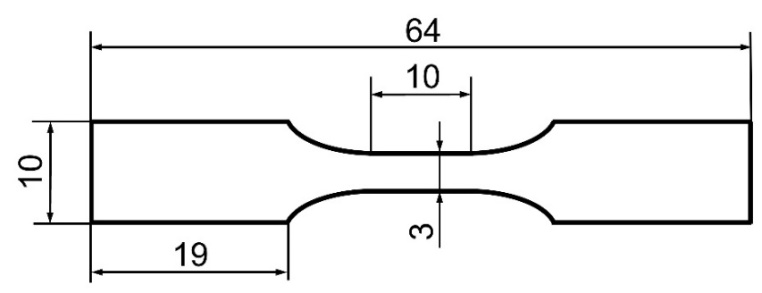


**Fig. S5.** Schematic of the ASTM type D dog-bone samples used for tensile testing. Dimensions are in mm and the specimen thickness is 3 mm.


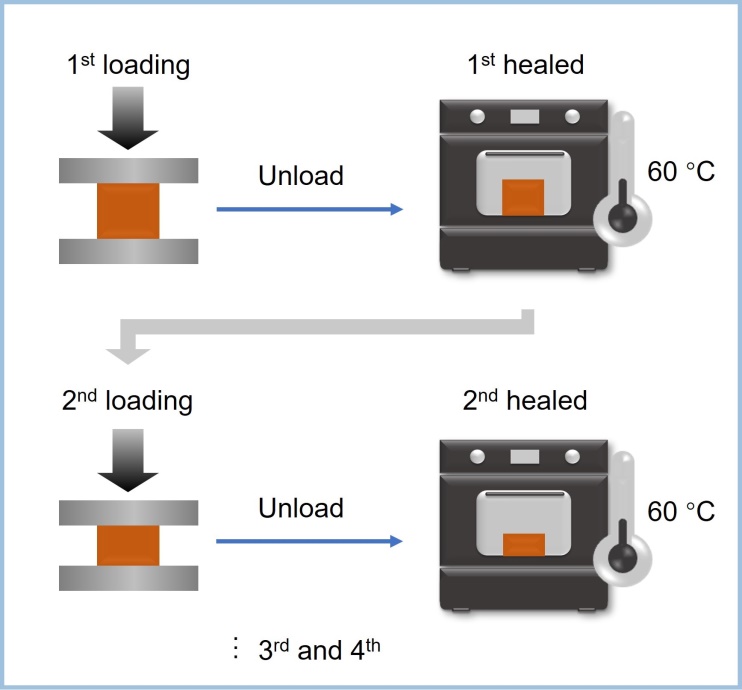


**Fig. S6.** Schematic illustrations of repeated loading and healing procedure of ACMO/MUA lattices.


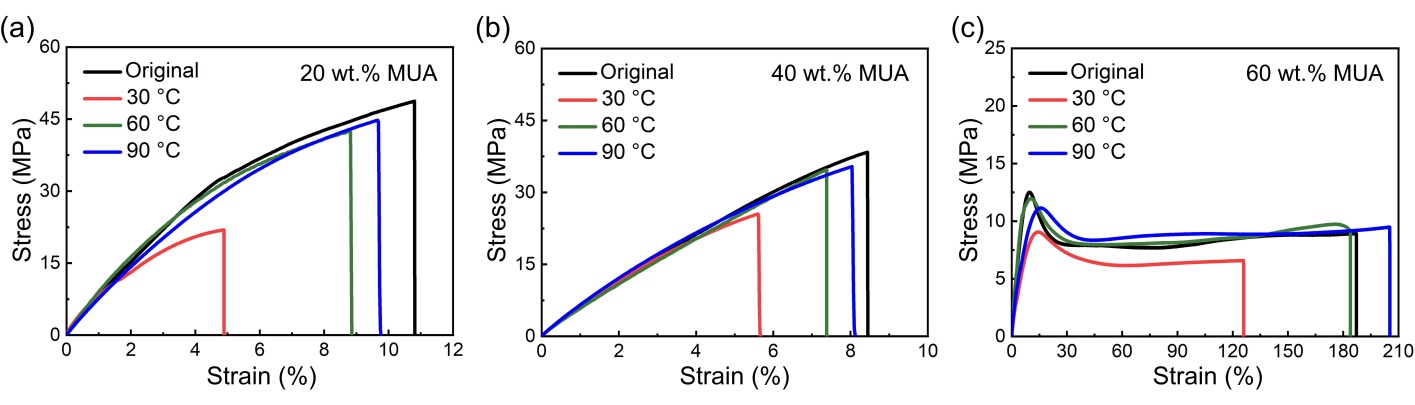


**Fig. S7.** Stress-strain curves of the composites with (a) 20 wt.%, (b) 40 wt.%, and (c) 60 wt.% MUA after healing at various temperatures for 12 h.


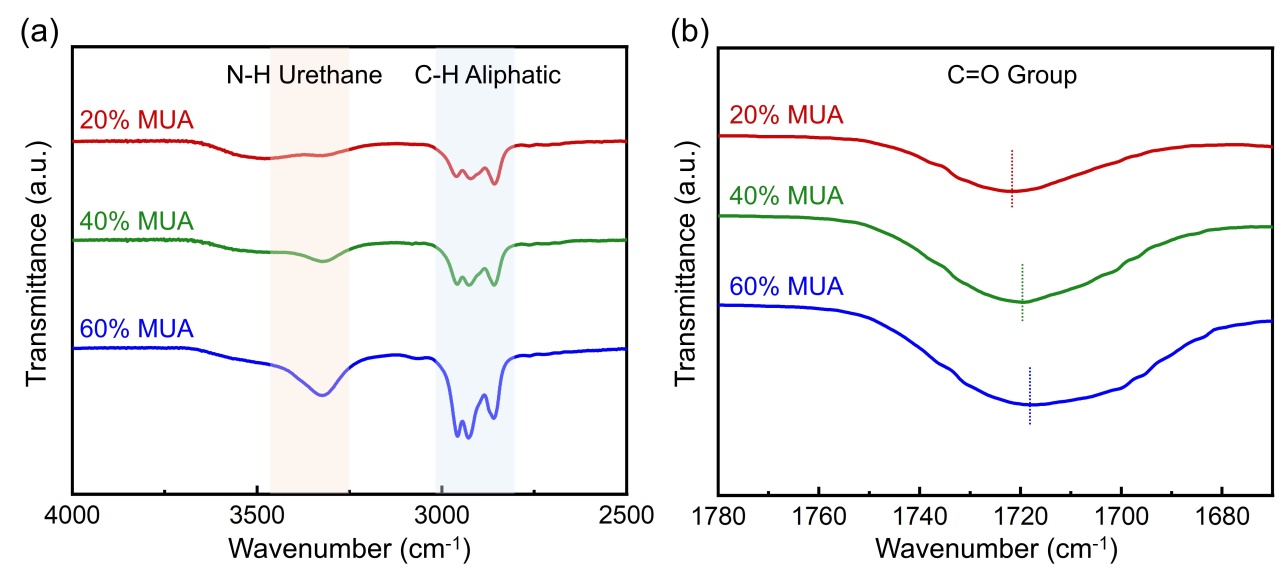


**Fig. S8.** FTIR spectra at the ranges of (a) 4000-2500 cm^-1^ and (b) 1780-1670 cm^-1^ of printed polymers with different MUA loadings (20 wt.%, 40 wt.%, and 60 wt.%).


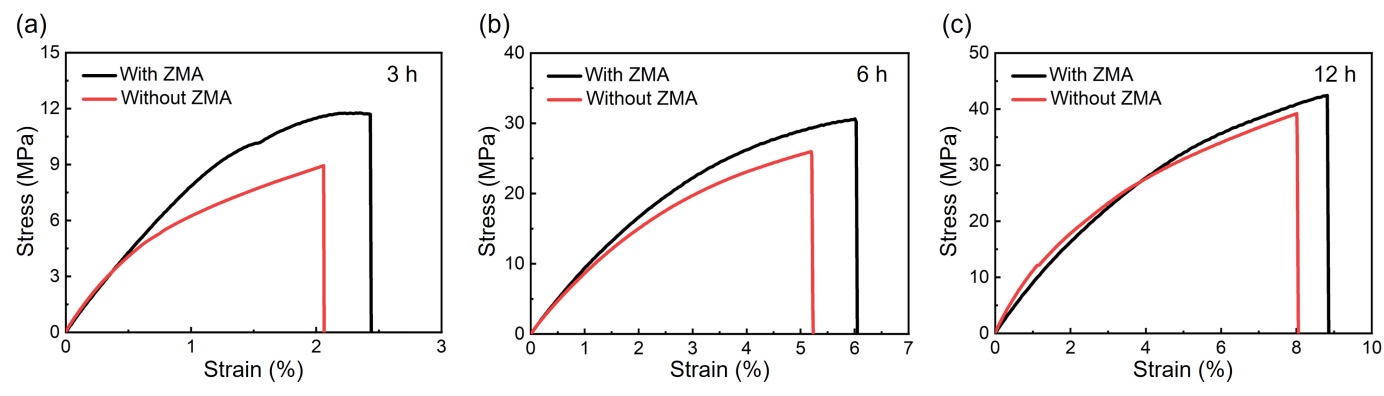


**Fig. S9.** Comparison of stress-strain curves of the composites with and without ZMA after healing at 60 °C for (a) 3 h, (b) 6 h, and (c) 12 h.


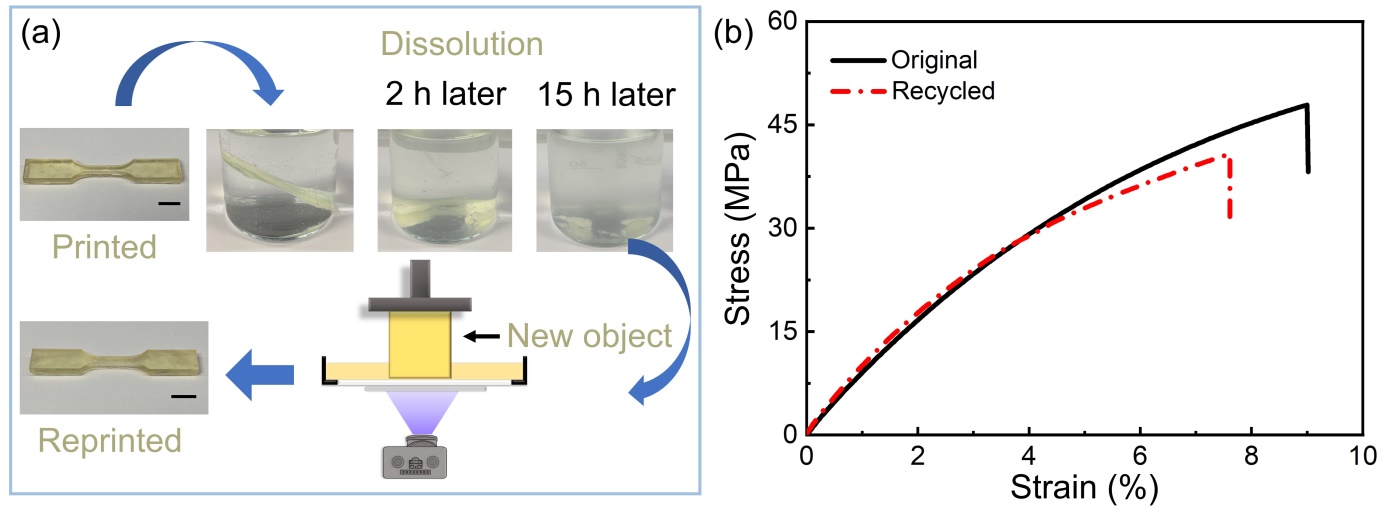


**Fig. S10.** The recyclability of the composites without ZMA. (a) The dissolution and reprinting process of printed 3D objects. (b) Stress-strain curves of initial and reprinted ACMO-MUA dog-bone samples with ACMO/MUA = 8:2. Scale bars are 1 cm.


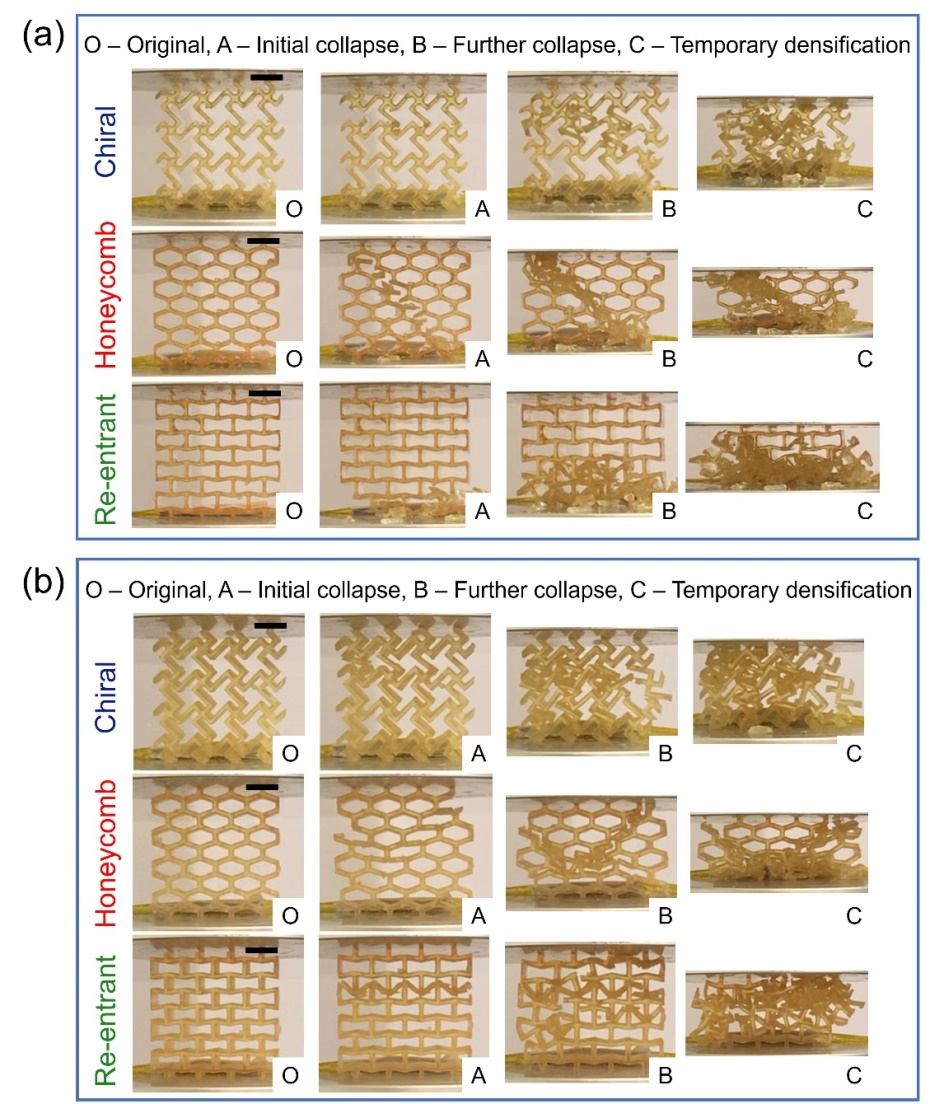


**Fig. S11.** Continuous compression tests of different cellular composites. Deformation behaviour at various stages of (a) neat ACMO and (b) ACMO/MUA lattices with chiral, honeycomb, and re-entrant cell topology. Scale bars are 1 cm.


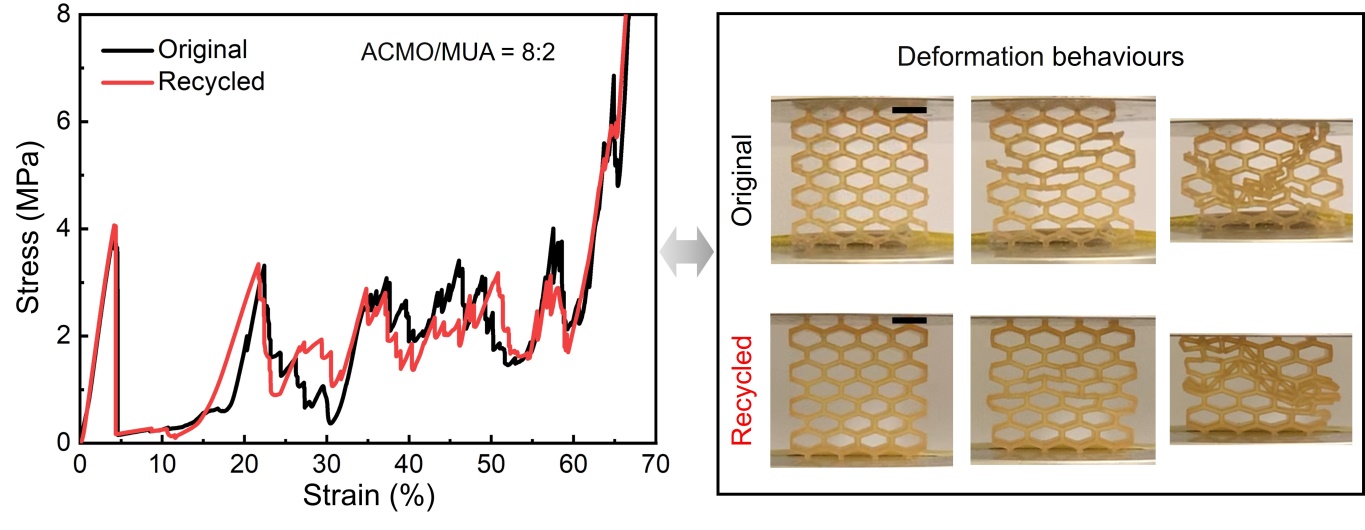


**Fig. S12.** Comparison of compressive stress-strain curves and deformation behaviours of the original and recycled ACMO-Zn-MUA lattices. Scale bars are 1 cm.


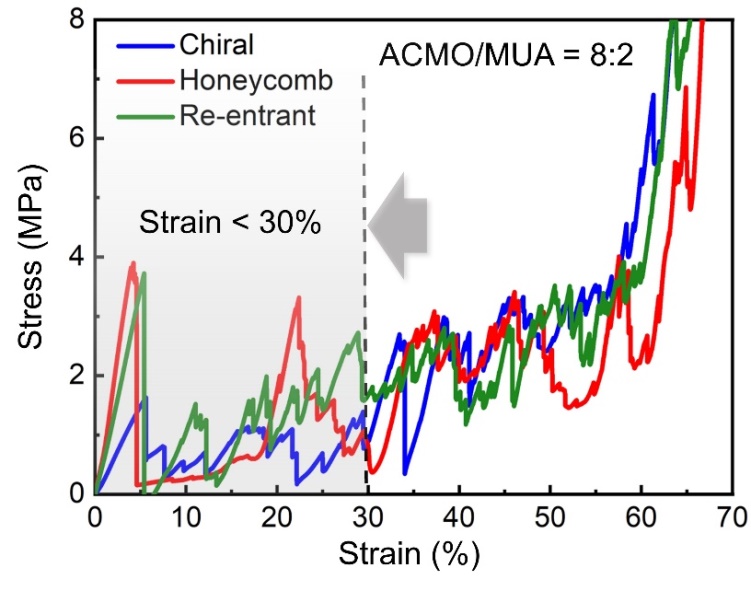


**Fig. S13.** Continuous compressive stress-strain curves of ACMO/MUA lattices within 30% strain as a control.


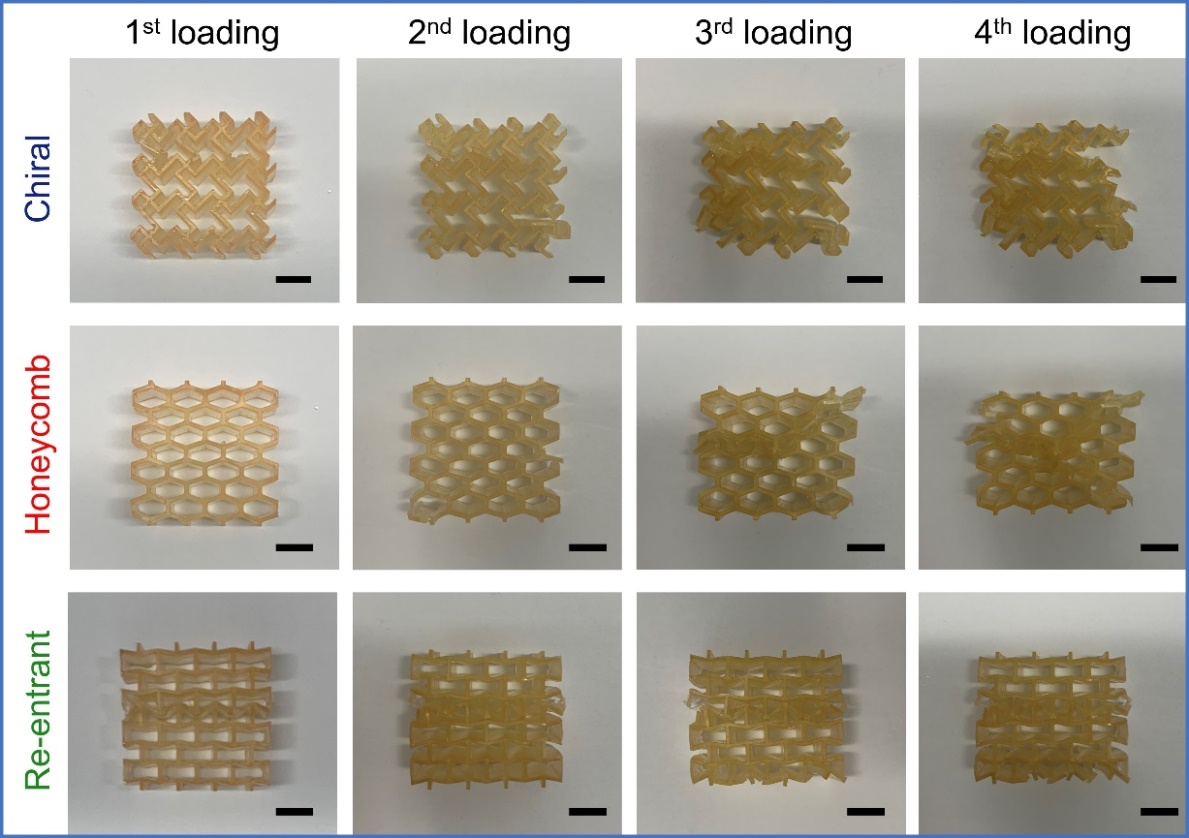


**Fig. S14.** Repeated loading-healing tests of different cellular composites. Deformation state of ACMO/MUA lattices with chiral, honeycomb, and re-entrant cell topology after different times of loading. Scale bars are 1 cm.


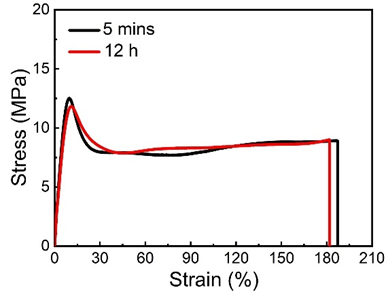


**Fig. S15.** Stress-strain curves of printed composites with 60 wt.% MUA after post-curing for different time.

**Table. S1.** Mechanical properties of original and healed ACMO-Zn-MUA composites after healing at 60 °C for various time.

| Samples |  | Young’s modulus  (MPa) | Tensile strength  (MPa) | Elongation at break  (%) | Toughness  (MJ·m^−3^) |
| --- | --- | --- | --- | --- | --- |
| 20 wt.% MUA | Original  3 h  6 h  12 h | 808.8  800.9  849.6  855.3 | 48.7  11.7  30.6  42.4 | 10.8  2.4  6.1  8.9 | 3.35  0.19  1.20  2.38 |
| 40 wt.% MUA | Original  3 h  6 h  12 h | 589.3  552.1  570.4  579.6 | 38.4  12.2  27.9  34.8 | 8.4  2.7  5.9  7.4 | 1.82  0.17  0.87  1.36 |
| 60 wt.% MUA | Original  3 h  6 h  12 h | 192.5  157.2  211.3  214.6 | 12.5  8.4  11.4  12.0 | 187.1  122.2  162.4  183.9 | 15.70  7.22  13.45  16.00 |

**Table. S2.** Mechanical properties of original and healed ACMO-Zn-MUA composites after healing at various temperatures for 12 h.

| Samples |  | Young’s modulus  (MPa) | Tensile strength  (MPa) | Elongation at break  (%) | Toughness  (MJ·m^−3^) |
| --- | --- | --- | --- | --- | --- |
| 20 wt.% MUA | Original  30 °C  60 °C  90 °C | 808.8  795.2  855.3  702.9 | 48.7  21.9  42.4  44.8 | 10.8  4.9  8.9  9.7 | 3.35  0.69  2.38  2.65 |
| 40 wt.% MUA | Original  30 °C  60 °C  90 °C | 589.3  546.4  579.6  614.8 | 38.4  25.5  34.8  35.4 | 8.4  5.6  7.4  8.0 | 1.82  0.82  1.36  1.65 |
| 60 wt.% MUA | Original  30 °C  60 °C  90 °C | 192.5  103.1  214.6  118.7 | 12.5  9.1  12.0  11.1 | 187.1  125.9  183.9  205.2 | 15.70  8.30  16.00  18.19 |

**Table. S3.** Mechanical properties of original and recycled ACMO-Zn-MUA composites.

| Samples | Young’s modulus  (MPa) | Tensile strength  (MPa) | Elongation at break  (%) | Toughness  (MJ·m^−3^) |
| --- | --- | --- | --- | --- |
| Original | 808.8 | 48.7 | 10.8 | 3.35 |
| Recycle 1 | 793.1 | 49.8 | 11.1 | 3.36 |
| Recycle 2 | 802.6 | 49.9 | 10.5 | 3.18 |
| Recycle 3 | 654.9 | 46.7 | 9.8 | 2.69 |

**Table. S4.** The maximum tensile strength, self-healing properties, and recyclability of UV curable polymers reported in the literature.

| 3D printable resins | Maximum tensile strength  (MPa) | Self-healing conditions /Strength recovery | Recyclability | References |
| --- | --- | --- | --- | --- |
| Maleic acid (MA)/choline chloride (ChCl)/acrylamide (AAm) | 0.7 | At room temperature for 24 h /55%~88% | - | [1] |
| 2-Hydroxyethyl methacrylate (HEA)/ polyurethane-based photopolymer resin (PUSA) | 11.5 | At 80 ºC for 12 h /17%~95% | - | [2] |
| 2-Hydroxyethyl methacrylate (HEA)/urethane acrylate/furan-maleimide Diels-Alder (fmDA) | 4.9 | At 110 ºC for 24 h + 80 ºC for 24 h /10%~99% | - | [3] |
| *κ*-carrageenan/polyacrylamide (PAAm) | 0.1 | At water bath temperature of 90 ºC for 5~40 min /35%~99% | - | [4] |
| 4-acryloylmorpholine (ACMO)/ carboxyl multi-walled carbon nanotubes (c-CNTs)/BYK | 18.7 | At room temperature for tens of minutes, under mild pressure (∼14 KPa) /<55% | - | [5] |
| Urethane monoacrylate (UMA)/acrylic acid (AA) | ~22.0 | At temperature of 30 ºC~90 ºC for 1~12 h /16%~99% | Hot pressing process | [6] |
| 4-acryloylmorpholine (ACMO)/isobornyl acrylate (IBOA) | >17.0 | - | Reprinting by the same DLP method | [7] |
| Vitrimers/UV curable solution system consisting of acrylate functional groups | ~55.0 | - | Grinding process + the same DLP method | [8] |
| 4-acryloylmorpholine (ACMO)/ monofunctional urethane (MUA) | 48.7 | At temperature of 60 ºC for 3~12 h /25%~95% | Reprinting by the same DLP method | Current work |

**Table. S5.** Comparison of the *T*_g_ of self-healing polymers reported in the literature.

| Self-healing temperature (^o^C) | Glass transition temperature, *T*_g_ (^o^C) | References |
| --- | --- | --- |
| 23 | 5.0~58.0 | [9] |
| 23 | - | [5] |
| 23 | -72.9 | [10] |
| 23 | -7.9~-6.4 | [11] |
| 23 | -89.4~-62.3 | [1] |
| 23/60 | -42.5~-21.6 | [12] |
| 35 | ~7.0 | [13] |
| 60 | -19.0~142.0 | Current work |
| 70 | -6.1~8.7 | [14] |
| 80 | -8.0~4.9 | [15] |
| 80 | -7.0~18.6 | [16] |
| 80 | 4.4~20.2 | [17] |
| 80 | - | [2] |
| 90 | 44.7 | [4] |
| 90 | -18.0~101.0 | [6] |
| 110 | 16.2~30.4 | [3] |

**Table. S6.** Toughness of neat ACMO and ACMO-Zn-MUA lattices with chiral, honeycomb, and re-entrant cell topology under continuous compression.

| Samples |  | | Toughness (MJ·m^−3^) |
| --- | --- | --- | --- |
| Neat ACMO | Chiral | 1.09 | |
|  | Honeycomb | 1.00 | |
|  | Re-entrant | 1.21 | |
| ACMO-Zn-MUA | Chiral | 1.24 | |
|  | Honeycomb | 1.31 | |
|  | Re-entrant | 1.48 | |

**Table. S7.** Toughness of ACMO-Zn-MUA lattices with chiral, honeycomb, and re-entrant cell topology under continuous and repeated compression.

| Samples |  | | Toughness (MJ·m^−3^) |
| --- | --- | --- | --- |
| Chiral | Continuous loading (corresponding to 2^nd^ loading)  2^nd^ loading | 0.0334  0.0292 | |
|  | Continuous loading (corresponding to 3^rd^ loading)  3^rd^ loading  Continuous loading (corresponding to 4^th^ loading) | 0.0765  0.0825  0.0477 | |
|  | 4^th^ loading | 0.0617 | |
| Honeycomb | Continuous loading (corresponding to 2^nd^ loading)  2^nd^ loading  Continuous loading (corresponding to 3^rd^ loading) | 0.0228  0.0462  0.0820 | |
|  | 3^rd^ loading  Continuous loading (corresponding to 4^th^ loading) | 0.1145  0.1173 | |
|  | 4^th^ loading | 0.0810 | |
| Re-entrant | Continuous loading (corresponding to 2^nd^ loading)  2^nd^ loading  Continuous loading (corresponding to 3^rd^ loading)  3^rd^ loading  Continuous loading (corresponding to 4^th^ loading)  4^th^ loading | 0.0341  0.0474  0.0308  0.0410  0.0690  0.0639 | |

**Supplementary videos**

**Video S1** Compression deformation behaviour of pure ACMO lattice with chiral topology

**Video S2** Compression deformation behaviour of pure ACMO lattice with honeycomb topology

**Video S3** Compression deformation behaviour of pure ACMO lattice with re-entrant topology

**Video S4** Compression deformation behaviour of ACMO-Zn-MUA lattice with chiral topology

**Video S5** Compression deformation behaviour of ACMO-Zn-MUA lattice with honeycomb topology

**Video S6** Compression deformation behaviour of ACMO-Zn-MUA lattice with re-entrant topology

**References**

[1] L. Cai, G. Chen, B. Su, M. He, 3D printing of ultra-tough, self-healing transparent conductive elastomeric sensors, Chem. Eng. J. (2021) 130545.

[2] X. Li, R. Yu, Y. He, Y. Zhang, X. Yang, X. Zhao, W. Huang, Self-healing polyurethane elastomers based on a disulfide bond by digital light processing 3D printing, ACS Macro Lett. 8(11) (2019) 1511-1516.

[3] A. Durand-Silva, K.P. Cortés-Guzmán, R.M. Johnson, S.D. Perera, S.D. Diwakara, R.A. Smaldone, Balancing self-healing and shape stability in dynamic covalent photoresins for stereolithography 3D printing, ACS Macro Lett. 10(4) (2021) 486-491.

[4] S. Liu, L. Li, Ultrastretchable and self-healing double-network hydrogel for 3D printing and strain sensor, ACS Appl. Mater. Interfaces. 9(31) (2017) 26429-26437.

[5] B. Guo, X. Ji, X. Chen, G. Li, Y. Lu, J. Bai, A highly stretchable and intrinsically self-healing strain sensor produced by 3D printing, Virtual Phys. Prototyp. 15(sup1) (2020) 520-531.

[6] G. Zhu, Y. Hou, J. Xiang, J. Xu, N. Zhao, Digital light processing 3D printing of healable and recyclable polymers with tailorable mechanical properties, ACS Appl. Mater. Interfaces. 13(29) (2021) 34954-34961.

[7] G. Zhu, Y. Hou, J. Xu, N. Zhao, Reprintable polymers for digital light processing 3D printing, Adv. Funct. Mater. 31(9) (2021) 2007173.

[8] H. Li, B. Zhang, R. Wang, X. Yang, X. He, H. Ye, J. Cheng, C. Yuan, Y.F. Zhang, Q. Ge, Solvent-free upcycling vitrimers through digital light processing-based 3D printing and bond exchange reaction, Adv. Funct. Mater. (2022) 2111030.

[9] Y. Yanagisawa, Y. Nan, K. Okuro, T. Aida, Mechanically robust, readily repairable polymers via tailored noncovalent cross-linking, Science. 359(6371) (2018) 72-76.

[10] L. Zhang, Z. Liu, X. Wu, Q. Guan, S. Chen, L. Sun, Y. Guo, S. Wang, J. Song, E.M. Jeffries, A highly efficient self-healing elastomer with unprecedented mechanical properties, Adv. Mater. 31(23) (2019) 1901402.

[11] M.W.M. Tan, G. Thangavel, P.S. Lee, Rugged soft robots using tough, stretchable, and self-healable adhesive elastomers, Adv. Funct. Mater. (2021) 2103097.

[12] Y. Peng, Y. Yang, Q. Wu, S. Wang, G. Huang, J. Wu, Strong and tough self-healing elastomers enabled by dual reversible networks formed by ionic interactions and dynamic covalent bonds, Polymer. 157(2018) 172-179.

[13] Y. Eom, S.-M. Kim, M. Lee, H. Jeon, J. Park, E.S. Lee, S.Y. Hwang, J. Park, D.X. Oh, Mechano-responsive hydrogen-bonding array of thermoplastic polyurethane elastomer captures both strength and self-healing, Nat. Commun. 12(1) (2021) 1-11.

[14] Y.-m. Ha, Y.-O. Kim, S. Ahn, S.-k. Lee, J.-s. Lee, M. Park, J.W. Chung, Y.C. Jung, Robust and stretchable self-healing polyurethane based on polycarbonate diol with different soft-segment molecular weight for flexible devices, Eur. Polym. J. 118(2019) 36-44.

[15] C. Zou, C. Chen, Polar-functionalized, crosslinkable, self-healing, and photoresponsive polyolefins, Angew. Chem. Int. Ed. 59(1) (2020) 395-402.

[16] J. Hu, R. Mo, X. Jiang, X. Sheng, X. Zhang, Towards mechanical robust yet self-healing polyurethane elastomers via combination of dynamic main chain and dangling quadruple hydrogen bonds, Polymer. 183(2019) 121912.

[17] Y. Song, Y. Liu, T. Qi, G.L. Li, Towards dynamic but supertough healable polymers through biomimetic hierarchical hydrogen‐bonding interactions, Angew. Chem. Int. Ed. 57(42) (2018) 13838-13842.
